# Collateral effects of the coronavirus disease 2019 pandemic on lung cancer diagnosis in Korea

**DOI:** 10.1186/s12885-020-07544-3

**Published:** 2020-10-29

**Authors:** Ji Young Park, Ye Jin Lee, Taehee Kim, Chang Youl Lee, Hwan Il Kim, Joo-Hee Kim, Sunghoon Park, Yong Il Hwang, Ki-Suck Jung, Seung Hun Jang

**Affiliations:** 1 Division of Pulmonary, Allergy and Critical Care Medicine, Department of Internal Medicine, Hallym University Sacred Heart Hospital, Hallym University Sacred Heart Hospital, 22 Gwanpyeong-ro 170 beon-gil, Dongan-gu, Anyang, 14068 Republic of Korea; 2 Division of Pulmonary, Allergy and Critical Care Medicine, Kangdong Sacred Heart Hospital, Hallym University, Seoul, Republic of Korea; 3 Division of Pulmonary, Allergy and Critical Care Medicine, Kangnam Sacred Heart Hospital, Hallym University, Seoul, Republic of Korea; 4 Division of Pulmonary, Allergy and Critical Care Medicine, Chuncheon Sacred Heart Hospital, Hallym University, Chuncheon, Republic of Korea

**Keywords:** COVID-19, Lung cancer, Delay, Diagnostics

## Abstract

**Background:**

The COVID-19 pandemic is predicted to significantly affect patients with lung cancer, owing to its rapid progression and high mortality. Studies on lung cancer diagnosis and treatment during an epidemic are lacking. We analyzed the impact of COVID-19 on lung cancer diagnosis in Korea, where lung cancer incidence continues to rise.

**Methods:**

The number of newly diagnosed lung cancer cases in three university-affiliated hospitals during the pandemic and their clinical features were compared with lung cancer cases diagnosed during the same period in the past 3 years. The effectiveness of measures taken by the study hospitals to prevent nosocomial transmission was reviewed.

**Results:**

A total of 612 patients were diagnosed with lung cancer from February through June, 2017–2020. During the pandemic, the number of patients who sought consultation at the division of pulmonology of study hospitals dropped by 16% from the previous year. Responding to the pandemic, the involved hospitals created physically isolated triage areas for patients with acute respiratory infection symptoms. Wide-range screening and preventive measures were implemented, thus minimizing the delay in lung cancer diagnosis. No patient acquired COVID-19 due to hospital exposure. The proportion of patients with stage III–IV non-small-cell lung cancer (NSCLC) significantly increased (2020: 74.7% vs. 2017: 57.9%, 2018: 66.7%, 2019: 62.7%, *p* = 0.011). The number of lung cancers diagnosed during this period and the previous year remained the same.

**Conclusions:**

The proportion of patients with advanced NSCLC increased during the COVID-19 pandemic.

**Supplementary Information:**

The online version contains supplementary material available at 10.1186/s12885-020-07544-3.

## Background

Severe acute respiratory syndrome coronavirus 2 (SARS-CoV-2), first discovered in Wuhan, China, spread throughout neighboring Asian countries and has become a global pandemic [1]. Aside from the social and economic impacts of coronavirus disease 2019 (COVID-19), the repercussions of this pandemic on the public health and healthcare systems cannot be fully assessed solely based on the number of cases and deaths declared by each country. According to a recent British report, approximately 12,000 additional deaths not associated with the virus occurred since the pandemic, compared with the previous year [2]. This suggests that patients with chronic and severe acute diseases were restricted from availing healthcare services. In the United States, the number of brain imaging tests performed to diagnose stroke dropped by 39% during the COVID-19 pandemic. Furthermore, a study reported that hospital admissions due to acute myocardial infarction declined by 48% [3, 4]. The incidence of out-of-hospital cardiac arrest reportedly increased [5]. Furthermore, there were concerns that cancer was underdiagnosed [6]. The decrease in the number of cancer diagnoses was observed and can be attributed to both presentational delay (the reduced number of patients seeking consult in healthcare facilities or referrals from primary clinics) and delays in the diagnostic process.

Our researchers were also able to see a decrease in the number of patients presenting to the hospital. The Korean government policy was to refrain from using the hospital unless a severe symptom developed; the continuous warnings through broadcasts potentially caused hesitations in necessary healthcare visits [7]. Cancer progression due to delayed diagnosis of lung cancer must reduce a patient’s chance of curative radical surgery. The study aimed to appropriately assess the impact of the COVID-19 pandemic on lung cancer diagnosis and indirectly assess the preventive measures taken by the involved hospitals in South Korea, where lung cancer incidence continues to rise. This study will provide pilot data for the development of preventive measures during the COVID-19 pandemic and other future epidemics of emerging infectious diseases.

## Methods

The epidemiology of patients with lung cancer was analyzed using the lung cancer cohorts at three teaching hospitals affiliated with Hallym University Medical Center [8, 9]. Each hospital is a 1000-bed healthcare facility. The process for lung cancer diagnoses at the hospitals included the first referral of patients suspected with lung cancer by primary healthcare facilities or health examination centers and initial presentation to the outpatient clinic or emergency department with their respiratory symptoms. During the COVID-19 pandemic, the hospitals implemented measures to prevent nosocomial transmission among patients and healthcare providers. All data were analyzed retrospectively.

On January 20, 2020, the first case of COVID-19 was confirmed in South Korea. Since then, national health authorities have responded to the rapid spread of the virus by raising the infectious disease alert level on January 27, 2020. Preventive measures such as banning entry from Hubei, China, since February, reinforced personal hygiene (universal masking), and social distancing practices were implemented. Therefore, we defined the period of collateral effect of the COVID-19 pandemic from February to June, when the national prevention policies were lowered. Newly diagnosed patients with lung cancer during this period were compared with patients diagnosed during the same period in the previous years. The inclusion criteria were: 1) patients, aged 18 years or older, who were diagnosed with pathological lung cancer between February and June during 2017–2020 and 2) presence of either small cell lung cancer (SCLC) or non-small cell lung cancer (NSCLC). The exclusion criteria were 1) metastatic cancer or uncontrolled cancer of a different organ, 2) recurrent lung cancer, 3) lymphoma, thymic cancer, and malignant pleural mesothelioma, and 4) radiological suspicion without pathologic diagnosis. To analyze the changes in availing healthcare services during the COVID-19 pandemic, we analyzed the weekly number of outpatients in the study hospital.

### Statistical analysis

The collected data were analyzed using SPSS Statistics for Windows, version 26.0 (IBM Corp., Armonk, NY, USA). Statistical analyses were performed using two-tailed *P* values < 0.05. Clinical features and relevant variables of patients with lung cancer diagnosed during the COVID-19 pandemic and patients with lung cancer diagnosed in previous years were compared using the chi-square test and t-test. The number of newly diagnosed patients with lung cancer was tallied in weekly intervals by year and month, and the changes in these numbers were comparatively analyzed with those of previous years. The number of patients by lung cancer subtype and stage was compared before and after the pandemic using the chi-square test or linear by linear association. The number of pulmonology outpatients was compared with that of the previous year using the same method.

## Results

Data of 169 patients with lung cancer diagnosed during the COVID-19 pandemic between February and June 2020 and 443 patients diagnosed between February and June during 2017–2019 were analyzed (Table 1). The mean age of the entire study population was 69.1 ± 10.7 years, and 69.3% were male. Histologically, 532 (86.9%) patients had NSCLC, while 80 (13.1%) had SCLC. There were no significant differences in age, performance status (ECOG), smoking history, and cancer subtypes between the two groups.

**Table 1:** Patient characteristics according to diagnoses years

	2020 (Feb–Jun) (*n* = 169)	2017–19 (Feb–Jun) (*n* = 443)	*P* value
Age at diagnosis, mean ± SD	69.4 ± 11.1	69.0 ± 10.6	0.637
Sex, Female	53 (31.4%)	135 (30.5%)	0.832
Performance, ECOG 0–2, (%)	91.7%	93.2%	0.686
Smoking, never smoker, (%)	34.4%	29.3%	0.443
Subtype			0.807
NSCLC	146 (86.4%)	386 (87.1%)	
SCLC	23 (13.6%)	57 (12.9%)	
Stage of NSCLC			0.015^*^
IA–IB	31 (21.2%)	98 (25.4%)	
IIA–IIB	6 (4.1%)	47 (12.2%)	
IIIA–IIIC	28 (19.2%)	76 (19.7%)	
IVA–IVB	81 (55.5%)	165 (42.7%)	
Stage of SCLC			0.042
Limited disease	11 (47.8%)	14 (24.6%)	
Extensive disease	12 (52.2%)	43 (75.4%)	

*SD* standard deviation, *ECOG* Eastern Cooperative Oncology Group, *NSCLC* non-small cell lung cancer, *SCLC* small cell lung cancer

^*^Statistical significance was tested by the linear by linear association

**Fig. 1:**
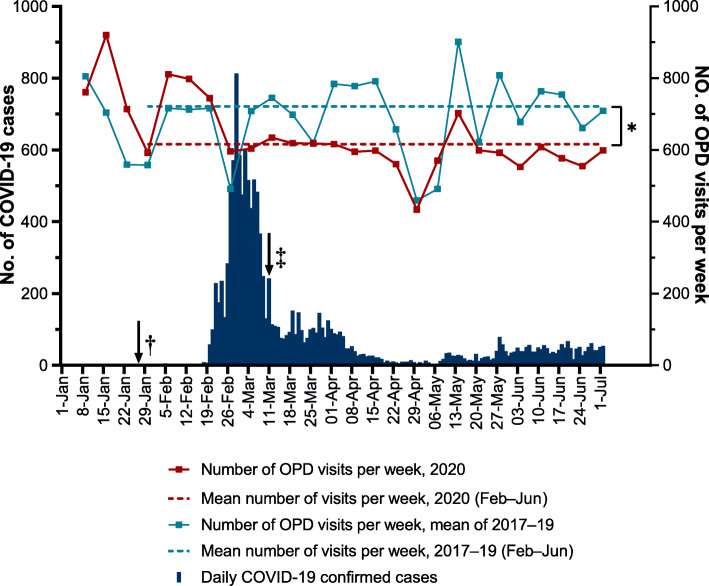
Daily numbers of coronavirus disease (COVID-19) cases in South Korea and weekly number of patients in the pulmonary outpatient clinics department (OPD) of study hospitals. *p < 0.001, †National infectious disease alert (from Level 2 to Level 3), ‡WHO announced COVID-19 is a pandemic

**Fig. 2:**
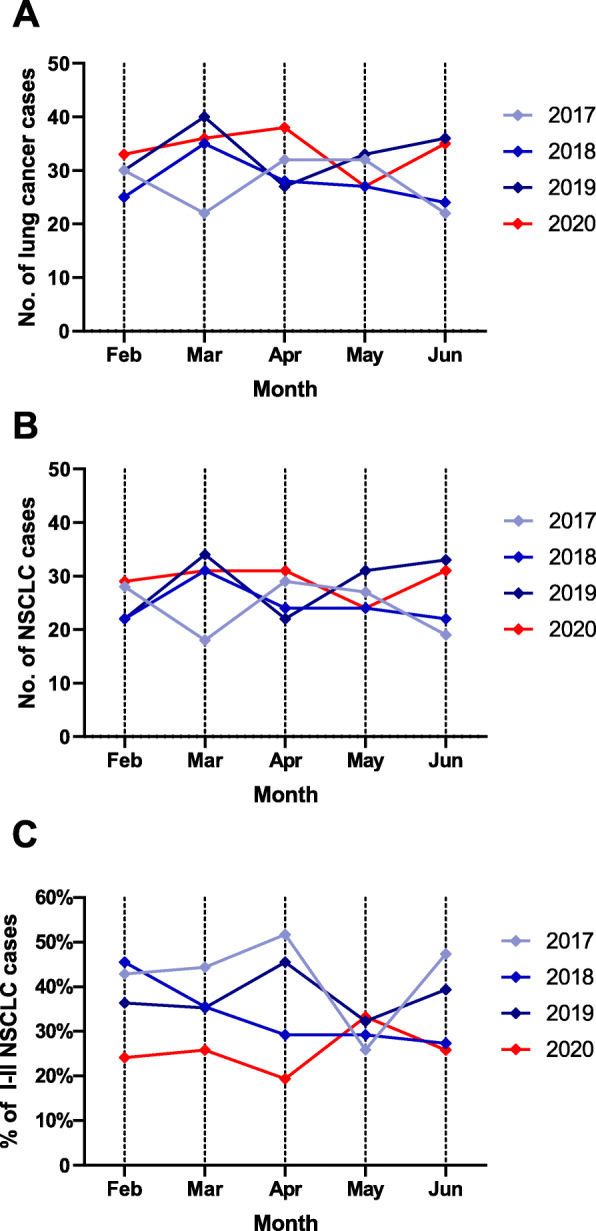
**a** Monthly number of lung cancer diagnoses, **b** monthly number of non-small cell lung cancer (NSCLC) diagnoses, **c** monthly number of stage I or II NSCLC by years

**Fig. 3:**
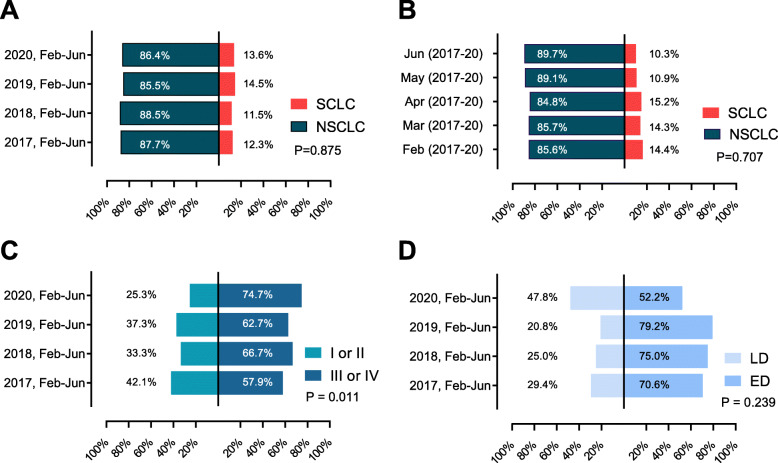
**a** Proportion of lung cancer subtypes by years (Feb–Jun), **b** lung cancer subtype by months (2017–2020), **c** stage of non-small cell lung cancer (NSCLC) by years (Feb–Jun), **d** small cell lung cancer (SCLC) stage by years (Feb–Jun). LD: limited disease, ED: extensive disease

Fig. 1 shows the trends in the daily number of confirmed COVID-19 cases in South Korea and the number of outpatients who presented to the pulmonology clinic of the study’s hospital. In South Korea, COVID-19 cases spiked from the third week of February. During this period, the number of pulmonology outpatients at the study hospital dropped by 16% from the previous year. The decline in the number of outpatients continued until June (weekly average number of patients: 721 during 2017–2019 vs. 616 in 2020, *p* < 0.001). Figure 2 shows the monthly number of new lung cancer diagnoses by year. There were no significant differences in the overall number of patients with lung cancer before and after the pandemic (2017: *N* = 138, 2018: *N* = 139, 2019: *N* = 166, 2020: *N* = 169, *p* = 0.605). There were no significant differences when the analysis was limited to NSCLC diagnoses only (2017: *N* = 121, 2018 *N* = 123, 2019: *N* = 142, 2020: *N* = 146, *p* = 0.437). Even with a decline in the number of outpatient visits, the number of new lung cancer diagnoses remained constant. There were also no differences in the percentage of histological subtypes by year and month (Fig. 3a and b).

During the COVID-19 pandemic, the proportion of stage III or IV cancer was 74.7%, which was significantly higher than that of the previous years (2017: 57.9%, 2018: 66.7%, 2019: 62.7%), while the proportion of stage I or II cancer decreased to 25.3% (*p* = 0.011) (Table 2 and Figure S1). The decline in the number of early lung cancer diagnosis was more evident during the early days of the pandemic (Fig. 2c). In the SCLC group, the proportion of patients with limited stage (47.8%) increased from that of the previous years, but the difference was not significant (2017: 29.4%, 2018: 25.0%, 2019 20.8%, 2020:47.8%, *p* = 0.239).

**Table 2:** Lung cancer stage stratified by subtypes and years

Lung cancer subtype	Stage		Total	*P* value^*^
NSCLC	Stage I–II	Stage III–IV		0.011
2017 (Feb–Jun)	51 (42.1%)	70 (57.9%)	121	
2018 (Feb–Jun)	41 (33.3%)	82 (66.7%)	123	
2019 (Feb–Jun)	53 (37.3%)	89 (62.7%	142	
2020 (Feb–Jun)	37 (25.3%)	109 (74.7%)	146	
Total (Feb–Jun)	182 (34.2%)	350 (65.8%)	532	
SCLC	Limited disease	Extensive disease		0.239
2017 (Feb–Jun)	5 (29.4%)	12 (70.6%)	17	
2018 (Feb–Jun)	4 (25%)	12 (75%)	16	
2019 (Feb–Jun)	5 (20.8%)	19 (79.2%)	24	
2020 (Feb–Jun)	11 (47.8%)	12 (52.2%)	23	
Total (Feb–Jun)	25 (31.3%)	55 (68.8%)	80	

*NSCLC* non-small cell lung cancer, *SCLC* small cell lung cancer

^*^Statistical significance was tested by the linear by linear association

### Preventive measures

In South Korea, prompt development and approval of the COVID-19 diagnosis kit enabled quick and wide-ranging screening since the early days of the pandemic. Screening was performed for those in direct contact with COVID-19 patients and those who showed symptoms of acute respiratory infection. Most university affiliated hospitals, including the hospitals in this study, have designated triage outpatient clinics and in-hospital wards as recommended by health authorities [10]. From the moment of presenting to the outpatient or emergency departments, patients with respiratory symptoms were seen by healthcare providers wearing personal protective equipment (PPE) in an isolated area. Suspected patients who needed to be hospitalized were admitted to an isolated ward until the test results were obtained. Even patients who yielded negative results were admitted to an isolated respiratory cohort ward. Patients who tested positive were provided continuous quarantined care or referred to national quarantine facilities. Patients admitted for lung cancer diagnosis, administration of anticancer agents, or surgery were admitted to a ward physically separated from the above-listed patients. They were required to undergo SARS-CoV-2 screening, and only those who tested negative were admitted. No hospital-acquired COVID-19 cases occurred in the hospitals.

While bronchoscopy is essential in the process of lung cancer diagnosis and staging, it is a high-risk, aerosol-generating procedure. Recently, bronchology societies published recommendations for the use of bronchoscopy during the pandemic [11]. Most guidelines recommend postponing elective bronchoscopy tests, but in the included hospitals, bronchoscopy was promptly performed on lung cancer-suspected patients. Although the guidelines recommend pre-test screening to identify those with fever, respiratory symptoms, and prior contact with COVID-19 patients, given that some COVID-19 patients are asymptomatic, uniform pre-bronchoscopy COVID-19 screening may be required in regions where community transmission of COVID-19 occurred [12]. In our study hospitals, patients admitted for lung cancer and outpatients with respiratory symptoms were required to undergo COVID-19 screening before bronchoscopy. None of the newly diagnosed patients with lung cancer in the study period were infected with COVID-19. To prevent the spread of infection within the hospital, only a number of healthcare professionals wearing PPEs performed the bronchoscopy in a well-ventilated negative-pressure room. Aerosol-generating procedures were not performed during the pretreatment or testing processes. After completing the test, surfaces were thoroughly disinfected. Percutaneous needle biopsy was also performed under similar preventive protocols.

## Discussion

In addition to the impact of the infection, collateral effects such as restricted healthcare access and service provision must also be taken into consideration. In the present study, we observed that the percentage of patients with more advanced stage cancers among newly diagnosed NSCLC patients during the pandemic was higher than that of previous years.

During the influenza H1N1 epidemic in 2009, a higher incidence of pneumonia and higher mortality were reported among patients with cancer, compared with that observed with the general population [13]. Nevertheless, there is a lack of studies examining the diagnosis of lung cancer and treatment guidelines during an epidemic. During the COVID-19 pandemic, several international societies presented guidelines for cancer diagnosis and treatments based on expert opinions [14]. However, these guidelines are not yet evidence-based. As there is still no effective drug or vaccine for COVID-19, it is difficult to anticipate when the pandemic will be eradicated. Meticulous evidence-based preparation is needed as another novel infectious disease may occur in the future [15].

The collateral effects of COVID-19 pandemic on the healthcare system affected both healthcare providers and patients. With a growing number of COVID-19 patients requiring hospitalization, the reallocation of human and other resources is a common phenomenon among healthcare facilities [16]. Consequently, clinical activities needed to diagnose and treat diseases, including cancer, will be hindered. In our study’s hospitals, personnel in the general cancer diagnosis areas were relocated to address the staff shortage for COVID-19 screening and care. A report on the impact of COVID-19 on the diagnosis of cancer showed that registration of new patients with cancer in the Netherlands national cancer registry dropped by about 25% between March and May 2020 [17]. In the United Kingdom, referrals of cancer-suspected cases decreased by about 80% [18, 19]. Patients are reluctant to visit a healthcare facility out of fear for infection. A survey on patients with lung cancer who participated in a clinical trial in Taiwan during the SARS outbreak reported that about 64% of the patients were reluctant to visit a hospital out of fear for infection, and about 4% of the patients decided to discontinue all treatment due to concerns of infection [20]. In fact, the decline in healthcare utilization may be only natural following media reports and study findings confirming local outbreaks of COVID-19 in healthcare facilities [21].

However, there is a problem that excessive concerns regarding COVID-19 beyond what is necessary could worsen the avoidance of healthcare facilities among patients with cancer and delay the necessary medical diagnosis and treatment. In the present study, the increased percentage of patients with stage III or IV cancer with a decreased percentage of patients with earlier stages of cancer in the NSCLC group suggested a presentational delay in the diagnosis of lung cancer. Patients in symptomatic stage I or II lung cancer may have been diagnosed at an advanced stage after disease progression due to presentational delay. In contrast, asymptomatic early-stage patients are mostly diagnosed through screening; it is postulated that the number of early diagnoses of lung cancer decreased due to a decline in the medical checkup rate. The longer the pandemic period, the more significant the impact can be expected. On the other hand, the percentage of patients with limited stage cancer increased, albeit statistically insignificant, in the SCLC group. Because symptom onset is more common with SCLC than with NSCLC, this result may be attributed to the possibility that these patients consulted at a healthcare facility early on owing to respiratory symptoms during the COVID-19 pandemic [22].

The COVID-19 pandemic also impacted the treatment process. Anticancer therapy or surgery was postponed or canceled. A modeling study that analyzed the impact of delayed cancer surgery due to the COVID-19 pandemic reported that a three-month and six-month delay of surgery decreased the anticipated life-years gain after surgery by 19 and 43%, respectively [23]. Moreover, the impact was greater among patients with lung cancer. A dilemma occurs when elective surgeries are postponed in adherence to physical distancing and reorganization of healthcare resources because it contradicts the goal of minimizing delays of curative surgeries. Likewise, when considering cytotoxic anticancer therapy, it is important to weight its benefits with the risk of infection due to immunosuppression. For advanced stage lung cancer, it is ideal to choose agents that could reduce inpatient hospitalization or outpatient clinic visits when choosing cytotoxic anticancer agents for palliative therapy. The clinical and radiological features of COVID-19 pneumonia may be difficult to differentiate from pneumonia during anticancer therapy or the pneumonitis during immunotherapy or targeted therapy [24]. As such, the threshold for COVID-19 screening should be lowered for patients with lung cancer currently undergoing treatment. Healthcare providers should consider prompt testing of these patients for COVID-19 based on their symptoms and radiologic findings even when they had no prior contact with a confirmed patient.

The mortality from SARS-COV-2 infection is higher among patients with cancer than in the general population. In a cohort study of 928 cancer patients confirmed with COVID-19 infection in the US, Canada, and Spain, the all-cause mortality rate was high at 13% [25]. Factors associated with mortality risk were age, male sex, number of comorbidities, poor performance status, smoking status, and active cancer status. However, history of surgery within 4 weeks and overall anticancer treatment status, including targeted therapy, cytotoxic therapy, and immunotherapy, were not associated with mortality risk. A study on 102 lung cancer patients diagnosed with COVID-19 also demonstrated that the severity and mortality of COVID-19 were related to patient-specific features (smoking, chronic obstructive pulmonary disease, and heart failure) rather than cancer-specific features (surgery and recent systemic treatments) [26]. Therefore, if COVID-19 is well contained within the hospital, and healthcare resources are utilized appropriately, hospitals should avoid delaying surgery of operable cancers except typical indolent cases even during a pandemic.

Our research has limitations. First, this study included a limited number of hospitals and patients. Second, long-term follow-up is required to evaluate the prognosis of patients with a delayed diagnosis of lung cancer. Some patients with slowly progressing lung cancer may be safe with late diagnosis. However, most patients progress rapidly, even with early lung cancer [27, 28]. Considering the study results in which the median overall survival was 9 months when not treated at stage I lung cancer, it is reasonable to evaluate the rapid stage shift during the five-month study period [29]. Third, the effects of SARS-CoV-2 infection on the diagnosis and treatment process of lung cancer were not evaluated. None of the newly diagnosed lung cancer patients included in this study was diagnosed with COVID-19.

## Conclusions

We observed the presentational delay of NSCLC diagnosis during the COVID-19 pandemic. However, the proactive triaging of suspected patients, aggressive COVID-19 screening, and timely lung cancer diagnosis prevented a decrease in the number of diagnoses. Although the mission to control COVID-19 pandemic is essential, national health authorities should prepare accurate assessments and countermeasures of any collateral effects, which may threaten the accessibility of diagnostic management for patients with cancer. Creating a safe healthcare system during this pandemic is essential for effective clinical service delivery to patients with serious diseases such as cancer.

## Supplementary Information

**Additional file 1 **: **Figure S1.** The weekly mean number of lung cancer diagnoses during the 5 months, 2017–2020 (A). The weekly number of lung cancer diagnoses during the 5 months in the COVID-19 pandemic in Korea (B).

## Data Availability

The dataset used and analysed during the present study is available from the corresponding author upon reasonable request.

## Abbreviations

COVID-19: Coronavirus disease 2019
NSCLC: Non-small cell lung cancer
SCLC: Small cell lung cancer
SARS-CoV-2: Severe acute respiratory syndrome coronavirus 2
PPE: Personal protective equipment
