# Supplementary material for: Collateral effects of the coronavirus disease 2019 pandemic on lung cancer diagnosis in Korea
Source: BMC Cancer. 2020 Oct 29;20:1040. doi: 10.1186/s12885-020-07544-3 (PMC7594984; doi:10.1186/s12885-020-07544-3)

**Supplementary information**

**Figure S1.** The weekly mean number of lung cancer diagnoses during the five months, 2017–2020 (A). The weekly number of lung cancer diagnoses during the five months in the COVID-19 pandemic in Korea (B).


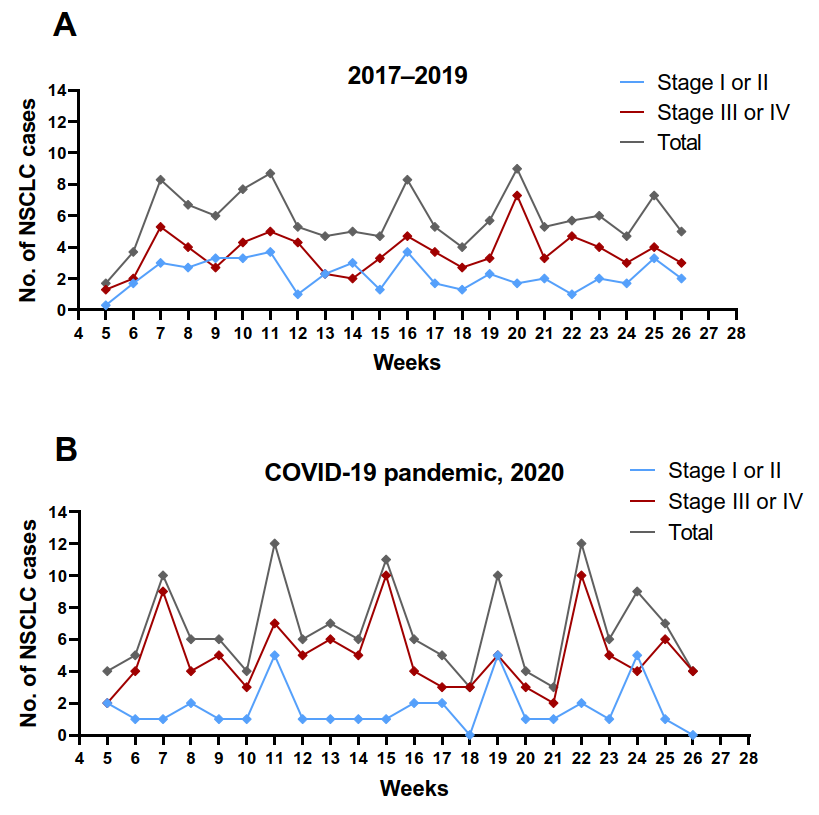

Supplement: Supplementary file 1 — Additional file 1 : Figure S1. The weekly mean number of lung cancer diagnoses during the 5 months, 2017–2020 (A). The weekly number of lung cancer diagnoses during the 5 months in the COVID-19 pandemic in Korea (B). [file 12885_2020_7544_MOESM1_ESM.docx]
